# Nurses’ Experiences of Providing Dysphagia Services Through the Internet+Nursing Service Care Model: Qualitative Study

**DOI:** 10.2196/67572

**Published:** 2025-06-30

**Authors:** Zhifang Ren, Ling Tong, Shuojin Fu, Shuai Jin, Yanling Wang, Qian Xiao

**Affiliations:** 1 School of Nursing, Capital Medical University, No.10 Xi-tou-tiao, You-an-men Wai, Feng-tai District, Beijing, 100069, China, 86 15210756682

**Keywords:** telehealth, dysphagia, telemedicine, nursing care, qualitative research, nursing, nurses, gerontology, geriatrics, older adults, aging, internet, content analyses, home health care

## Abstract

**Background:**

With China’s aging population and increasing prevalence of chronic diseases, the Internet+Nursing Service has emerged as a new care model, enabling registered nurses from medical institutions to provide home-based care through a web-based application and offline service model. This care model is particularly beneficial for vulnerable populations, such as patients with dysphagia, who face significant risks like malnutrition and aspiration pneumonia. Nurses play a critical role in delivering these services, yet their experiences, challenges, and support needs remain underexplored. Understanding these factors is essential for improving service quality and establishing standardized care guidelines.

**Objective:**

This study aims to explore the experiences and challenges of nurses providing the dysphagia-related Internet+Nursing Service, offering insights to guide the standardization and sustainable development of this innovative care model.

**Methods:**

A qualitative study was conducted with 18 nurses who had been providing the Internet+Nursing Service for patients with dysphagia for over 6 months. Purposive sampling ensured the selection of participants with relevant expertise. Semistructured interviews were used for data collection, focusing on nurses’ experiences, challenges, and recommendations. Data were analyzed using conventional content analysis, following an inductive approach to identify recurring themes and patterns.

**Results:**

The analysis revealed 3 key themes: value representation and social impact; nursing resources and staffing; and safety and management support. Nurses emphasized that patient-centered nursing services enhanced their sense of professional fulfillment and helped alleviate pressure on hospital nursing resources. However, challenges such as insufficient time and energy, inadequate manpower, and underexploitation of service potential limited service effectiveness. To ensure sustainability, nurses highlighted the need for standardized service processes, regular experience exchange, and stronger hospital involvement in managing and supporting the Internet+Nursing Service.

**Conclusions:**

This study highlights both the opportunities and challenges of delivering the dysphagia-related Internet+Nursing Service. While nurses acknowledge the value of this care model, addressing staffing shortages, improving training programs, and strengthening regulatory frameworks are essential for optimizing service delivery. Policy makers and health care institutions should develop standardized guidelines and supportive policies to enhance service sustainability and accessibility.

## Introduction

Amid rapid population aging and the increasing number of patients with chronic diseases in China, the demand for personalized and convenient nursing services has been steadily rising [1,2]. In 2019, the National Health Commission of China launched a pilot program in 6 provinces and cities, including Beijing and Shanghai, to evaluate the feasibility and optimize the implementation of the Internet+Nursing Service [3]. The Internet+Nursing Service is an emerging care model in which registered nurses from medical institutions provide home-based nursing to discharged or mobility-impaired patients [3]. This model uses a web-based platform and offline service mechanism, supported by internet and digital technologies. In this model, nurses voluntarily deliver care during designated off-duty hours and are compensated according to the specific service item and travel distance. The service fees paid by patients are determined locally under national policy guidelines and reflect labor value, transportation, and material costs [3]. Patients are required to upload relevant medical documents (eg, diagnostic and treatment records) when registering on the platform. Service orders may specify preferred time slots or designated nurses. Service requests are dispatched to nurses via a mobile application for acceptance or rejection. If no nurse is specified, the system assigns a qualified nurse based on availability and expertise. Once the order is accepted, the nurse provides the requested service at the patient’s home. At present, the Internet+Nursing Service is not uniformly included in the national health insurance coverage. Although patients generally pay out of pocket at present, some regions have begun piloting the inclusion of selected service items in their medical insurance reimbursement schemes [4]. Furthermore, commercial insurance products—such as long-term care insurance—may offer partial cost coverage for these services [5].

The Internet+Nursing Service connects patients with nurses via digital platforms, primarily offering home-based nursing care for patients with stable chronic conditions, such as urinary catheter changes, gastric tube replacements, and pressure ulcer care [3,6]. By integrating technology with professional nursing care, this model reduces logistical barriers, facilitates real-time progress tracking, and enhances follow-up care [6,7]. Furthermore, it bridges the gap between hospitals and home-based care, offering a seamless continuum of services [8]. This model not only alleviates the strain on health care resources but also enhances the accessibility of nursing care [9,10]. Given these demonstrated benefits, the National Health Commission has issued a policy directive to expand this pilot program nationwide, with standardized implementation protocols to ensure service quality and patient safety [6].

Dysphagia is a collection of signs and symptoms, mainly characterized by difficulties in the formation and transport of food masses [11]. Around 80% of dysphagia cases are associated with neurological or other underlying diseases, such as stroke, Parkinson disease, esophageal cancer, etc [11-13]. Previous studies have associated dysphagia with multiple adverse outcomes, including malnutrition, frailty, dehydration, respiratory infections, aspiration pneumonia, increased readmission rates, and even death [11,14]. Considering these severe outcomes, providing effective care and rehabilitation guidance is essential to improve patient outcomes and quality of life [11,15]. However, with shorter hospital stays, patients with dysphagia and their caregivers receive insufficient guidance and lack the knowledge to care for them in a busy clinical setting [16]. As a result, dysphagia patients represent a high-demand group for specialized nursing care, emphasizing the importance of extending nursing services to the home environment [17]. The Internet+Nursing Service model offers a promising solution for addressing the complex care needs of dysphagia patients by providing home-based, personalized nursing services. Due to their specialized care needs and heightened risk for adverse outcomes, dysphagia patients represent an ideal population for evaluating the implementation effectiveness of the Internet+Nursing Service [18]. Insights gained from this patient group, characterized by complex and intensive care requirements, may provide valuable lessons for adapting and expanding these services to other high-demand chronic patient populations.

The implementation of the Internet+Nursing Service places considerable demands on nurses, who must balance technical competence with interpersonal communication while adjusting to the home environment [19]. Since hospital responsibilities remain their priority, nurses can only provide home-based care during off-duty hours. This constraint, compounded by nationwide nursing shortages, heavy workloads, and stressful work environments, contributes to high rates of occupational burnout [19-21]. Understanding nurses’ perspectives is therefore crucial to identifying systemic challenges and informing practical strategies for sustainable service delivery. Despite its growing implementation, little research has examined nurses’ subjective experiences, role transitions, and coping strategies in delivering services. These experiences directly affect care quality and service outcomes [22], particularly in high-need populations such as patients with dysphagia. This lack of evidence limits our ability to fully understand the challenges and support needs in practice. Further qualitative inquiry is therefore essential to support long-term integration and improvement of this care model.

This study investigates the subjective experiences, key challenges, and coping strategies of nurses delivering care to patients with dysphagia through the Internet+Nursing Service care model. By addressing a theoretical gap in this underexplored area, the research deepens understanding of how nurses navigate care delivery in high-need home-based settings. The findings offer practical implications for optimizing nurse training and support systems and may inform the development of similar services for other vulnerable populations.

## Methods

### Study Design

A qualitative study was conducted with data collected through semistructured interviews with nurses between May and September 2023. This approach was chosen for its effectiveness in capturing the complex, context-specific, and emotional experiences inherent to nurses’ roles in a new care model—areas difficult to explore through quantitative methods [23]. This study adhered to the Consolidated Criteria for Reporting Qualitative Research (COREQ) guidelines to ensure transparency and rigor in reporting [24]. A completed 32-item COREQ checklist mapping to the manuscript content is provided as Checklist 1.

### Participants and Setting

Purposive sampling was used to recruit nurses who had experience delivering the Internet+Nursing Service. Eligible participants were those who had provided care to patients with dysphagia for a cumulative duration of more than 6 months. To enhance the diversity of the sample, participants were selected based on variation in service items (eg, nasogastric tube placement, feeding guidance, rehabilitation training, and nutritional monitoring), duration, gender, and geographic location. Participants were recruited from 4 regions—Beijing, Guangdong, Zhejiang, and Jiangsu—where the service was first piloted and has since been widely implemented. Participants were contacted via WeChat through hospital administrators. The researcher introduced the purpose and content of the study and provided detailed information to ensure participants’ full understanding. They were informed that participation was voluntary and that they could decline or withdraw at any time without any consequences. Informed consent was obtained prior to scheduling the interview.

### Data Collection

Based on the research purpose and a review of relevant literature, the interview guide was developed under the guidance of a nursing professor (QX). To provide further clarity, exploratory questions were added to the main interview questions to better capture the depth and complexity of participants’ experiences, as shown in Table 1.

**Table 1.** Interview guide.

Main question	Exploratory questions
Can you briefly talk about your thoughts on the Internet+Nursing Service?	What do you think are the main advantages of this care model? What challenges do you foresee in implementing this model?
How was your Internet+Nursing Service experience?	Can you share a specific example of a memorable experience? How do you feel this model has impacted your nursing practice?
Do you have any difficulties in providing the Internet+Nursing Service to people with dysphagia? Please expand on this.	What types of difficulties are most common? How do these difficulties affect your workflow or interactions with patients? Can you share a specific example of a challenging situation and how you handled it?
What areas would you like help and support with? Please expand on this.	What kind of help or support do you think would best solve the issues you encountered? Are there specific skills or resources you think you lack?
What do you think can be done to better serve people with dysphagia?	Are there any technologies or tools you believe would improve this service? What changes in the current process could enhance patient outcomes?

All participants were interviewed alone by the first author, a female PhD candidate with formal training in qualitative research, through Tencent Meeting, a secure digital conferencing platform. Given her familiarity with the topic, she consciously minimized potential bias by adhering closely to the interview guide and maintaining a neutral stance throughout data collection.

The first interview was conducted with a Chief Nurse who had extensive frontline experience in delivering the Internet+Nursing Service to patients with dysphagia. Her insights into practical challenges and strategies helped refine the focus of subsequent interviews. Interviews were video recorded, but for participants who declined to use their cameras due to privacy concerns, only audio was recorded. The interviews were conducted under private and secure conditions to ensure confidentiality and comfort for the participants. Most participants joined from their homes, a setting that was perceived as comfortable and conducive to open discussion. When participants mentioned their experiences and perceptions about service, the researcher encouraged them to elaborate and provide specific examples. Field notes were taken during the interviews to capture key observations such as participants’ tone, pauses, and non-verbal cues. These notes provided additional contextual insights to complement the interview transcripts, helping to enhance data interpretation. Each participant was interviewed once; no repeat interviews were conducted.

Data saturation was reached after interviewing the 16th nurse, and 2 additional interviews were conducted to confirm saturation and ensure data robustness. All 18 invited nurses agreed to participate, and none withdrew from the study. The average interview duration was 41.2 (range 38.5‐45.3) minutes.

### Data Analysis

All interviews were transcribed verbatim within 24 hours and analyzed concurrently with data collection. The transcripts were reviewed for consistency and returned to participants for member checking. Although no revisions were made, this step helped ensure accuracy and credibility. Data were analyzed using conventional content analysis, a method suitable for areas with limited theoretical and empirical foundations—such as nurses’ experiences in providing the Internet+Nursing Service for patients with dysphagia [25]. This approach allows categories to emerge inductively from the data, enabling a nuanced understanding grounded in participants’ perspectives without imposing preconceived frameworks [23,24,26].

Two authors (ZR and LT) independently conducted the initial data analysis. They first read the transcripts to become familiar with the content, then separately identified significant statements and conducted open coding. This process yielded 203 initial codes, reflecting the depth and diversity of the participants’ responses. Similar codes were grouped into categories and subcategories. Some codes specific to dysphagia-related experiences were incorporated into broader thematic structures to ensure conceptual coherence. When a disagreement arose between the 2 analysts, 2 experts in qualitative nursing research (QX and YW) were asked to arbitrate in order to ensure the scientific validity of the coding. Finally, themes and subthemes were developed based on the categories and subcategories[27]. Data coding and analysis were conducted manually. All interviews were carried out and analyzed in Chinese. Results were subsequently translated into English. The translation was reviewed by bilingual researchers to preserve the original meaning and contextual accuracy. While transcripts were returned to participants for accuracy checking, the findings were not returned for feedback.

### Ethical Considerations

This study was approved by the Medical Ethics Committee of the Capital Medical University (approval no. Z2022SY029). Although participants’ identities were known to the researcher during data collection, all data were deidentified prior to analysis by assigning unique identification codes. Informed consent was obtained from all participants, who were informed that their participation was voluntary and that they could withdraw from the study at any time without any consequences. Participants received a compensation of approximately US$30 in recognition of their time and contribution.

## Results

### Participants

In total, 18 nurses participated in this study, identified by codes N1-N18. In total, 17 (94.4%) were female and 1 (5.6%) was male. Participants’ ages ranged from 32 to 51 years, with a mean of 38.8 (SD 5.9) years and a median of 37.5 years(IQR: 33.0–44.3). More than half of the participants held the professional title of nurse practitioner-in-charge. All participants had attained an undergraduate degree. The duration of service involvement (in months) ranged from 6 to 60, with a mean of 30.1 (SD 14.6) months and a median of 25.0 months (IQR: 18.0–43.5), as shown in Table 2.

**Table 2.** Characteristics of the participants (N=18).

Number	Sex	Age (years), n	Title	Educational attainment	Length of participation (months), n
N1	Female	51	Chief nurse	Undergraduate	26
N2	Female	33	Nurse practitioner	Undergraduate	18
N3	Female	33	Nurse practitioner-in-charge	Undergraduate	18
N4	Female	37	Nurse practitioner-in-charge	Undergraduate	24
N5	Female	33	Nurse practitioner-in-charge	Undergraduate	15
N6	Female	47	Nurse practitioner-in-charge	Undergraduate	22
N7	Male	32	Nurse practitioner-in-charge	Undergraduate	48
N8	Female	38	Associate chief nurse	Undergraduate	40
N9	Female	32	Nurse practitioner	Undergraduate	48
N10	Female	40	Nurse practitioner-in-charge	Undergraduate	24
N11	Female	42	Nurse practitioner-in-charge	Undergraduate	42
N12	Female	45	Associate chief nurse	Undergraduate	36
N13	Female	34	Nurse practitioner-in-charge	Undergraduate	48
N14	Female	37	Nurse practitioner-in-charge	Undergraduate	6
N15	Female	45	Associate chief nurse	Undergraduate	26
N16	Female	44	Associate chief nurse	Undergraduate	60
N17	Female	34	Nurse practitioner-in-charge	Undergraduate	22
N18	Female	41	Nurse practitioner-in-charge	Undergraduate	18

### Overview of the Findings

**Figure 1.**
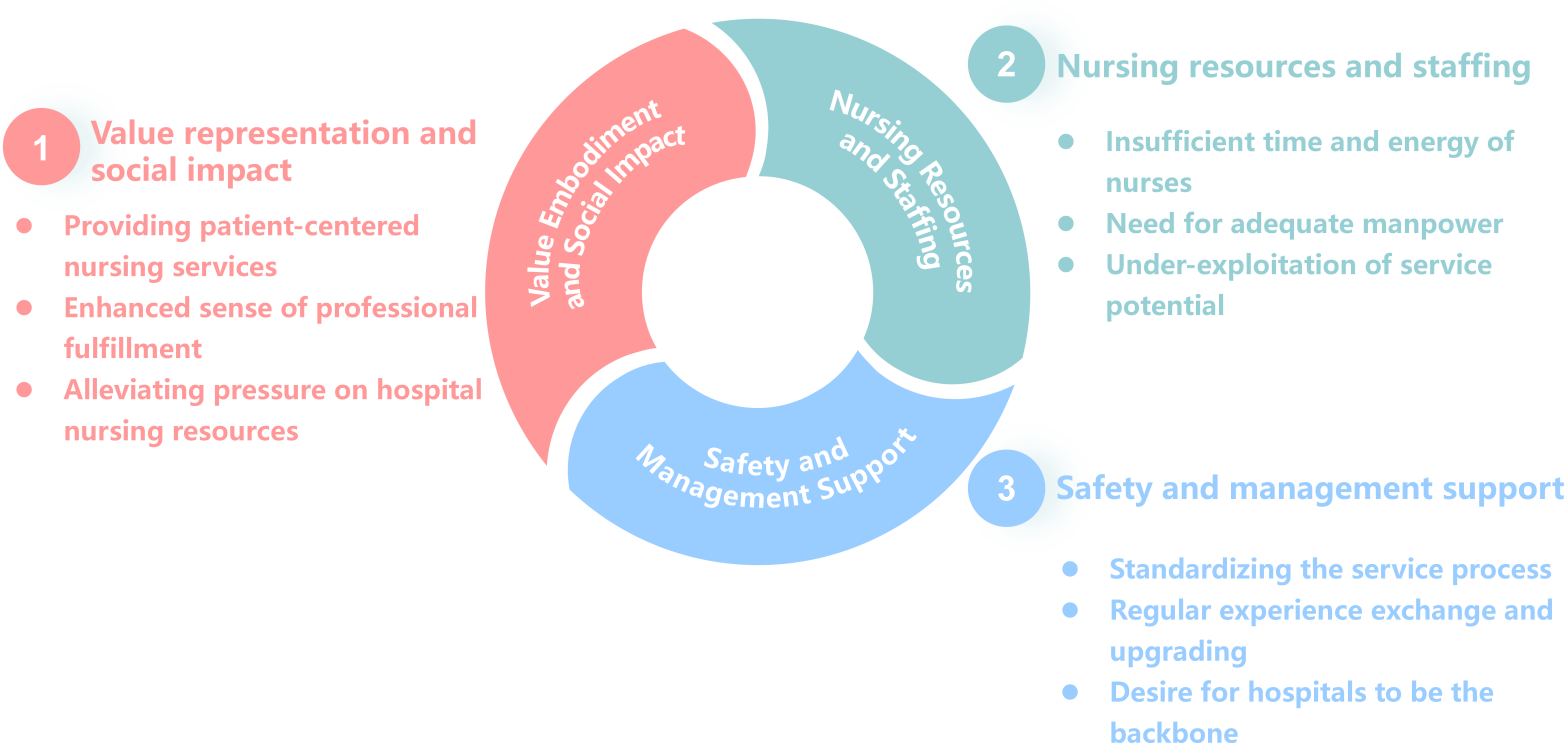
Overview of themes and subthemes.

A total of 3 main themes and 9 subthemes were identified through data analysis. The overall coding framework, including the hierarchical relationship between themes and subthemes, is presented in Figure 1. Participant quotations were used to support each theme, ensuring consistency between the raw data and the analytical findings.

### Value Representation and Social Impact

#### Providing Patient-Centered Nursing Services

Nurses emphasized that health services should be patient-centered, with patient safety as the highest priority. Before providing home care, nurses carefully assess each patient’s physiological condition, medical history, and risk of potential emergencies. Patients considered unsuitable for home-based care are excluded from service provision to mitigate safety risks.

*Our goal is to assist patients in resolving their issues effectively. We need to consider and address all possible situations to prevent any negative outcomes for the patient, ourselves, or the hospital.*

[N9]

Many nurses noted that dysphagia often results from underlying conditions, such as Alzheimer disease or stroke. These patients are typically discharged once their primary illness is under control, but they are not fully adjusted to their condition. Additionally, family members frequently lack adequate knowledge about care, particularly in managing feeding tubes, which adds complexity to post-discharge care. The continuity of care is insufficient. However, with the implementation of the Internet+Nursing Service, nurses have more time and energy to offer personalized care, educate family members, and provide comprehensive nursing services that address the gaps left by in-hospital care.

*In our neurology department, numerous patients are discharged unconscious, and their families are at a loss regarding patient care and feeding tube management. We are overwhelmed in the hospital, responsible for ten patients each, making it hard to provide detailed care instructions. Internet+Nursing Service can effectively make up for this shortcoming.*

[N15]

*Post-discharge continuity of care in our hospital is not well-executed due to strained human resources and limited economic benefits, leading to a lack of motivation. With Internet+Nursing Service, we now have more time to assist patients and guide their families, significantly improving patients’ quality of life.*

[N1]

Conducting home visits enables nurses to develop deeper empathy, gain insights into family challenges, and better understand the underlying factors influencing patient and caregiver behaviors. This improves compliance and effectiveness of health promotion efforts, thereby enhancing patient satisfaction.

*Visiting patients’ homes, observing their environment, and interacting with their families make us realize the challenges of caregiving. This empathy helps us understand family frustrations.*

[N7]

*In the hospital, families might not pay full attention to our advice, but at home, they handle patient care alone and lack guidance. They value our home visits, listening attentively and following our instructions.*

[N10]

#### Enhanced Sense of Professional Fulfillment

Participation in the Internet+Nursing Service offers nurses both spiritual rewards and economic benefits, reinforcing their sense of professional value.

*After years of clinical work, one might feel numb, but home visits rekindle that early career passion. I feel I can significantly impact patients’ lives beyond administering needles and medications.*

[N1]

Nurses who participated in home-based services feel their skills and values are more fully used.

*The home environment differs greatly from the hospital. Some equipment and supplies need improvement, so we designed a clinic bag with a countertop and even applied for a patent.*

[N8]

#### Alleviating Pressure on Hospital Nursing Resources

Nurses dedicate their off-duty hours to delivering home care, thereby optimizing nursing resources and alleviating hospital burdens. This reduces the strain on hospital staff and allows them to focus on critical cases. This model enhances the efficiency of resource allocation while ensuring timely, personalized care for patients, particularly those with chronic conditions or follow-up needs. Nurses emphasized that the Internet+Nursing Service serves as an effective extension of hospital care, bridging gaps in resource limitations and improving health care accessibility.

*Sometimes a simple issue like feeding difficulty can be resolved with a bit of water, but families don’t understand and rush to the hospital, exacerbating the strain on hospital resources. With Internet+Nursing Service, they can place an order for us to visit, eliminating the need for a hospital trip.*

[N4]

### Nursing Resources and Staffing

#### Insufficient Time and Energy of Nurses

Nurses experience significant pressure while working in hospitals, characterized by high work intensity, heavy responsibilities, and often long, continuous shifts, which result in inadequate personal time and energy. After completing their shifts, nurses lack the time and additional energy required to engage in the Internet+Nursing Service. Despite their willingness to provide these services, maintaining a work-life balance often limits their ability to do so.

*It’s been a week of work, I’m having a hard time taking a day off, I want to rest at home and spend time with my child.*

[N11]

*I am reluctant to participate in the Internet+Nursing Service because in a tertiary hospital like ours, nursing manpower is very scarce in itself, and if we have to arrange for two more nurses to go out, the department has to be even busier...Together with the time spent on transportation, preaching, and instruction, I can only change one gastric tube in a morning, which is too long.*

[N12]

#### Need for Adequate Manpower

Nurses emphasized that effective delivery of the Internet+Nursing Service requires more than ad hoc task allocation; it necessitates a comprehensive system encompassing operations, management, and service provision. Assigning this responsibility on a part-time basis may lead to reduced accountability and uncertainty regarding the service’s future development. Furthermore, each nurse in the hospital already has specific roles and responsibilities, making it difficult to effectively manage additional duties related to the Internet+Nursing Service. Presently, the Internet+Nursing Service encounters challenges including inadequate understanding of market demand and weak home quality control.

*The ideal situation would be for an independent department to be set up in the future to provide us with follow-up support, for example, if we encounter any difficulties during the service, we know who to go to and who can help us solve the problem!*

[N16]

#### Underexploitation of Service Potential

Although many nurses across hospitals are qualified to provide the service, relatively few are actively engaged. In some cases, no nurses are available to respond to service requests. This situation results in a significant underutilization of the nursing workforce and hinders the further development of the Internet+Nursing Service. Several factors contribute to the limited utilization of the service’s potential. First of all, the talent pool is insufficient. There are many nurses with service qualifications in the hospitals, but the leaders, in order to fully guarantee the quality of service, prioritize nurses with more adequate service experience on the basis of national standards, resulting in other nurses who are willing to do so being unable to take orders. Secondly, the service motivation of nurses is low. Some nurses said that the salary of the Internet+Nursing Service is too low, which does not match with their contribution. Third, the service becomes inefficient when patients live far from the hospital. Although transportation subsidies are provided, the time spent commuting is not compensated. In some cases, nurses spend up to 4 hours on a single round trip and can only serve 1 patient per day, which they report as unsustainable and physically exhausting.

*Let’s say there are five people in our unit who can provide the service, but only two of them make frequent visits. If several orders come in at the same time, how can we ensure timely service? Relying solely on those two individuals would compromise service quality, as they may become fatigued and need rest. For instance, if a gastric tube is dislodged, it cannot wait until the next day-it requires immediate attention.*

[N7]

*It’s 200 dollars for most of the day’s work, and it’s hot and tiring and mentally tight. I’d rather not have the money.*

[N3]

*I just thought if I could get a closer order, the patient could be closer to my home, but catching up with the traffic jam I have to travel almost 3 hours just to get there, it’s too slow.*

[N8]

### Safety and Management Support

#### Standardizing the Service Process

Nurses mentioned during the interviews that the current service process lacks standardization to some extent, which is crucial for ensuring service safety. For instance, in the current process, nurses use real-time voice recording and positioning functions, including detailed data during the indwelling of gastric tubes. However, nurses are solely responsible for recording and filling in these data. They express concerns about whether these records would serve as evidence to protect them in case of safety issues.

*After the operation, we will have records, such as how long the lower gastric tube was left in place, whether there is any abnormality in the implantation process, and then whether the pipeline is smooth, whether the fixation is good and so on, and then after the record is completed, it is best to confirm with the family and then sign. Otherwise, we only have one nurse, and the authenticity of the operation record will be questioned.*

[N8]

In addition, nurses express skepticism about the effectiveness of safety and security measures. The platform is equipped with features such as 1-button alarms and real-time recordings to protect nurses’ safety. However, nurses question whether these systems can accurately recognize incidents at a patient’s home and whether police assistance can be summoned promptly in case of personal injury.

*I know that we are given insurance, police and other measures, but if something does happen, it might be late. Because I haven’t encountered this, I don’t know how long it will take for the police to come...Even if the insurance pays me money, I still want the protection of health and safety more.*

[N14]

There is disorder in the management of consumables. Typically, consumables are uniformly provided by a third-party platform, but these items obtained through this channel are non-reimbursable and more expensive. In some regions, hospitals allow family members to use the patient’s health insurance card to buy supplies like gastric tubes at a discounted rate, while others do not permit this. Some patients choose to purchase consumables themselves through e-commerce platforms, where ensuring quality becomes challenging. To ensure consumable safety, certain regions have started to restrict patients from buying their own supplies. Nurses often face difficulties addressing patients’ inquiries and doubts in such situations.

*Sometimes the platforms have the option of self-purchasing consumables and sometimes they don’t. Families will ask us why we were able to buy our own before and now we can’t. Is it because we are making money out of the middle. These are questions that are hard for us to explain*

[N4]

*In itself, the Internet+Nursing service is for the convenience of the patient, if the family members are allowed to prescribe consumables when they come to the hospital, the service loses its property of convenience*

[N6]

#### Regular Experience Exchange and Upgrading

Nurses wish to engage in regular exchange of experiences, particularly across regions, to share insights and prevent mistakes, thereby enhancing overall nursing standards.

*For example, in our city, all hospitals work together to open an Internet-based summary meeting for experience exchange, although it is said that you are doing very well, but maybe people may do better, the exchange is necessary, we take the strengths and complement the weaknesses. We also hope to send people outside to learn, because you stay in the original position for a long time, you think you are one of the best, then you do not know that outside, a hundred flowers bloom.*

[N5]

Nurses often have suggestions for platform improvements during their use and desire these to be effectively communicated to platform technicians. Therefore, nurses are eager to participate in regular communication meetings where they can share their experiences and needs directly with platform service staff.

*I hope there will be meetings with the platform and then we nurses can have a few representatives who can give us feedback for enhancement and improvement.*

[N12]

#### Desire for Hospitals to be the Backbone

While providing services, nurses often experience anxiety and concern, particularly regarding potential accidents that could impact patient safety, their professional reputation, and the hospital’s image. Nurses expressed their expectation that the hospital would act as a strong backbone, offering not only emotional and professional support but also taking responsibility in cases of disputes. They hoped the hospital would advocate for them in public and provide clear policies, legal protections, and timely assistance when issues arise. This reassurance would help alleviate their stress and empower them to focus on delivering high-quality care with confidence.

*When I go to a patient’s home to provide care, they might not know my name, but they definitely know which hospital I work for. If I don’t do a good job, they’ll think poorly of the hospital too. When I’m out there, I represent the hospital’s reputation. So, I have to give 100% effort, but it does make me feel quite stressed.*

[N8]

*I’m really worried about what would happen if there’s a dispute during the service or if the patient is dissatisfied. I’m concerned about how my supervisor would respond and handle the situation. I don’t want to risk losing my formal job because of Internet+Nursing Service—it’s just a part-time role for me.*

[N4]

## Discussion

### Overview

This qualitative study identified 3 key themes: value representation and social impact, nursing resources and staffing, and safety and management support. The findings suggest that nurses perceive their participation in the Internet+Nursing Service as a means of affirming their professional value; however, they face constraints related to time and energy. Additionally, concerns regarding nurse safety and potential nurse-patient disputes emerged as significant issues. Future efforts should focus on safeguarding nurses’ rights, addressing their concerns, and fostering the sustainable development of these services.

### Findings

In terms of value realization, nurses believed that the Internet+Nursing Service could provide patient-centered health services. Its implementation involves safety considerations that are distinct from in-hospital settings. Since nurses assume full responsibility for out-of-hospital care, patient safety became a central focus in their assessments [28]. Specifically, they assessed whether the patient’s underlying condition was suitable for home-based care, as well as potential emergency scenarios [29]. This safety-first approach aligns with evidence from international home health care contexts, where studies from Saudi Arabia highlight nurses’ strong competencies in creating safe environments for elderly care [30], and Iranian research emphasizes continuous risk assessment as fundamental to safe home health care delivery [28]. Nurses observed that patients who required regular gastric tube replacements often forgot their scheduled appointments. Creating a health record with automated reminders could help patients manage their gastric tube changes more effectively. Digital health interventions, such as short message reminders, have demonstrated significant potential in improving patient adherence to scheduled health care activities and associated health outcomes. This finding aligns with Tesfahun et al [31], who emphasized the role of short messages in enhancing health service utilization and outcomes. Furthermore, nurses noted that patients were often not fully prepared to manage their condition before discharge, and caregivers often lacked appropriate caregiving knowledge. This preparation gap has significant clinical implications, as studies have shown that 90% of patients were discharged with at least 1 unresolved health care issue [32], and being unready for discharge increased the risk of 30day unplanned readmission and mortality [33]. The Internet+Nursing Service can address this deficiency. After living at home for a period, patients can consult one-on-one with the nurse about problems they have not mastered, and nurses found that the effect of health education and guidance is better than in the hospital. Evidence from Israel’s Community-Based Home Hospitalization model confirms the advantages of community-based follow-up, demonstrating reduced hospital readmission rates, lower daily health care costs, and shorter lengths of stay compared to conventional inpatient care [34]. Moreover, when nurses enter the patients’ homes and learn about their economic and social environment, they can better understand the emotions or decisions of patients and their families. This improved understanding helps nurses empathize with the families and may reduce conflicts between nurses and patients. Empathy is widely recognized as a key element of home care services and is strongly associated with care satisfaction for both providers and recipients [35]. In Australian home care, stakeholders emphasized the importance of empathy and person-centered relationships for high-quality, individualized care [35]. These findings suggest that the Internet+Nursing Service improves care delivery and strengthens nurse-patient relationships, thereby promoting more holistic and person-centered care in community settings.

Nurses believed that providing the Internet+Nursing Service enhanced their sense of professional fulfillment. In traditional clinical settings, nurses primarily followed medical directives passively, lacking autonomy and experiencing low professional satisfaction. Consistent with previous studies, limited autonomy has been identified as a risk factor for nurse burnout [36]. In contrast, home-based services offered a stronger sense of achievement, as nurses received both patient gratitude and monetary recognition for their professional skills. This aligns with findings that highlight salary as a key contributor to nurses’ professional fulfillment [37]. Moreover, the home environment provided a new operational space compared to hospitals, allowing nurses to broaden their practice and apply their scientific skills, such as developing patents. This is crucial for advancing nurses’ professional growth [38]. Caring for patients with dysphagia involves considerable uncertainty, and caregivers often seek hospital confirmation due to concerns, straining health care resources. Providing targeted guidance on caregiving practices can help caregivers alleviate this burden and reduce unnecessary reliance on institutional care. Prior studies have demonstrated that transitional care interventions from hospital to community play a critical role in reducing readmission rates and emergency department visits [39]. Such transitional care support not only mitigates system-level pressures but also strengthens family caregiving capacity, thereby disrupting the discharge-readmission cycle. The urgent demand for services from patients and their families, along with the personal value expression of nurses, are positive influencing factors for nurses participating in the Internet+Nursing Service.

The shortage of nurses has long been a concern for health care delivery systems worldwide [40,41]. According to the World Health Organization (WHO) report, the global shortage of nurses based on population health needs is projected to reach 5.7 million by 2030 [42]. The Internet+Nursing Service model is no exception, facing similar human resource challenges. Nurses reported experiencing high levels of stress due to demanding work schedules and limited personal time. They often prioritize rest and family time, which reduces their motivation to accept assignments, consistent with findings from previous studies [43]. Nurses in this study expressed dissatisfaction with their salaries, citing a perceived misalignment between their compensation and the overall cost of service provision—a finding consistent with previous research [42,44]. Nurses noted that the time spent on home-based services, commuting, health education, and rehabilitation instruction exceeds that of equivalent tasks performed in hospitals. This mismatch between effort expended and remuneration received diminishes their motivation to accept assignments [45]. Furthermore, nurses highlighted in interviews that while many join the Internet+Nursing Service System, only a small fraction actively accept assignments. This suggests that the service’s full potential has yet to be realized. The reluctance of some nurses to take assignments increases the workload for those who do, increasing service burden and exacerbating human resource challenges within the Internet+Nursing Service. In contrast, Australia’s home-based nursing services are primarily provided by full-time hospital-assigned teams composed of experienced nurses. In comparison, China’s model depends on nurses volunteering during their off-duty hours, which may limit the consistency and sustainability of service delivery [46]. Currently, management responsibilities related to the Internet+Nursing Service are often delegated to managers who have other duties, which hinders effective patient demand assessment and service quality control. To address these issues, medical institutions and platforms must enhance collaboration, establish robust operational systems, cultivate a larger pool of service-oriented professionals, and improve practices related to recruitment, training, assignment dispatch, and quality assurance. Despite the ongoing challenges in the service process, participants remain committed to providing care because they recognize the significance of their work for both patients and caregivers.

Nurses consistently voiced concerns about safety, particularly personal safety, which aligns with previous findings [37]. Although the Internet+Nursing Service offers security measures, nurses reported ongoing uncertainty about emergency response protocols. In addition, nurses are solely responsible for assessing and documenting dysphagia patients, which raises concerns about the reliability and accountability of clinical records. Nurses emphasized the importance of sharing experiences through regular peer communication and expressed a strong desire to establish direct contact channels with service platforms [47]. Such channels would enable them to propose suggestions and contribute to service improvement effectively.

Nurses involved in the Internet+Nursing Service express concerns about potential medical disputes stemming from errors, which could negatively impact their routine hospital responsibilities [46,47]. Consequently, nurses seek support from hospitals, including both organizational and telematic support. Organizational support is crucial in how individuals perceive management’s attention and care, while telematic support ensures timely access to information and medical resources, such as health care teamwork [48,49].

### Future Directions

In the future, it will be essential to implement robust safety protocols—such as establishing standardized response times for emergency services (eg, police arrival times after alarm activation) and developing clear guidelines for insurance coverage (eg, compensation details and claim processing timelines)—to effectively address nurses’ concerns regarding safety and support. Moreover, establishing multifaceted communication platforms—such as professional conferences, web-based forums, and community media engagements—will be essential for fostering experience sharing among nurses and amplifying awareness of successful cases. This, in turn, will enhance their confidence in delivering services.

Research indicates that health care collaboration and organizational support are critical drivers for improving nursing service quality [50,51]. In this context, the creation of a web-based medical expert network could empower nurses by providing access to remote doctor consultations and real-time guidance during emergencies or complex clinical situations. Future policy making and management efforts should focus on safeguarding nurses’ rights, addressing their concerns, and optimizing their working conditions. Accelerating the development of the Internet+Nursing Service and improving delivery efficiency can help ensure that nurses’ efforts translate into measurable improvements in patient outcomes and health system performance.

### Limitations

This study has several limitations. First, it captured nurses’ experiences during a specific phase of Internet+Nursing Service implementation in selected regions. As service models and policy support continue to evolve, the transferability of these findings to other settings or later stages may be limited. Future research could explore how regional differences and policy developments shape nurses’ experiences over time. Second, nurses with favorable views or positive experiences may have been more likely to agree to participate, introducing potential selection bias. Third, the recruitment strategy did not specifically target nurses who had withdrawn from the Internet+Nursing Service; perspectives from this group could further elucidate reasons for disengagement. Finally, although participants verified their transcripts for accuracy, the aggregated findings were not returned in order to safeguard participant privacy, which may have limited additional verification of our interpretations.

### Conclusions

This study underscores the significant value of the Internet+Nursing Service in advancing patient-centered care, enhancing nurses’ professional fulfillment, and optimizing health care resource allocation. However, the findings also highlight critical concerns among nurses, particularly regarding safety, workload, and fair compensation, which require urgent attention. To fully harness the potential of the Internet+Nursing Service, the following measures are recommended:

First, establish standardized safety protocols, including clear emergency response mechanisms and comprehensive insurance coverage. Second, develop diversified communication and collaboration platforms to facilitate experience sharing and peer support among nurses. Third, enhance organizational support by integrating remote medical resources to provide real-time guidance and safeguard nurses’ well-being. Fourth, optimize service operations and refine human resource management to ensure both efficiency and quality.

By implementing these strategies, not only can nurses’ job satisfaction and sense of security be strengthened, but patients will also receive more efficient and high-quality care. Ultimately, these efforts will contribute to the sustainable development of the Internet+Nursing Service and provide a solid foundation for continued innovation and progress in the health care sector.

## Supplementary material

10.2196/67572 Checklist 1 COREQ (Consolidated Criteria for Reporting Qualitative Research) 32-item checklist

## Abbreviations

COREQ: Consolidated Criteria for Reporting Qualitative Research

## References

[1] Javorszky SM, Reiter R, Iglseder B (2023). Validation of a Geriatric Bedside Swallowing Screen (GEBS): protocol of a prospective cohort study. JMIR Res Protoc.

[2] Pike K, Moller CI, Bryant C, Farrow M, Dao DP, Ellis KA (2023). Examination of the feasibility, acceptability, and efficacy of the online personalised training in memory strategies for everyday program for older adults: single-arm pre-post trial. J Med Internet Res.

[3] Notice of the General Office of the National Health Commission on carrying out the pilot work of Internet + Nursing Service [Web page in Chinese]. The State Council of the People’s Republic of China.

[4] Internet + Nursing patients enjoy professional services at home [Web page in Chinese]. Ningbo Municipal People’s Government.

[5] Internet+Nursing to promote the sustainable development of ageing in place [Web page in Chinese]. Economic Reference News.

[6] Circular of the General Office of the National Health Commission on further promoting the pilot work of ‘Internet + Nursing' Service [Web page in Chinese]. The Official Website of the Chinese Government.

[7] Huang R, Xu M, Li X, Wang Y, Wang B, Cui N (2020). Internet-based sharing nurse program and nurses’ perceptions in China: cross-sectional survey. J Med Internet Res.

[8] Ge Y, Xu F, Yuan H, Tang A, Dong L, Ren P (2022). Qualitative study on participation willingness of nursing staff in Internet+Nursing Service [Article in Chinese]. Soft Science of Health.

[9] Du J, Lun X, Zhao L, Li H (2024). Analysis on the practical effects of ‘Internet + Nursing Service’ in Shandong province [Article in Chinese]. Chin Nurs Manage.

[10] Hu Y, Li Y, Luo S, Zou T, Ding M (2024). Construction of an "Internet+Traditional Chinese Medicine nursing" service capability evaluation index system based on the three-dimensional quality structure model [Article in Chinese]. Chin J Nurs.

[11] Labeit B, Michou E, Hamdy S (2023). The assessment of dysphagia after stroke: state of the art and future directions. Lancet Neurol.

[12] Cnossen IC, van Uden-Kraan CF, Rinkel R (2014). Multimodal guided self-help exercise program to prevent speech, swallowing, and shoulder problems among head and neck cancer patients: a feasibility study. J Med Internet Res.

[13] Gonçalves IMP, Pontes-Silva A, Zica MM, Barasuol AM, Maciel E da S, Quaresma FRP (2023). Profile of oropharyngeal dysphagia patients in a teaching hospital in Northern Brazil: a descriptive cross-sectional study. Rev Assoc Med Bras (1992).

[14] Yang RY, Yang AY, Chen YC, Lee SD, Lee SH, Chen JW (2022). Association between dysphagia and frailty in older adults: a systematic review and meta-analysis. Nutrients.

[15] Chen L, Xiao LD, De Bellis A (2016). First-time stroke survivors and caregivers’ perceptions of being engaged in rehabilitation. J Adv Nurs.

[16] Carlsson E, Ehnfors M, Eldh AC, Ehrenberg A (2012). Accuracy and continuity in discharge information for patients with eating difficulties after stroke. J Clin Nurs.

[17] Yang CP, Cheng HM, Lu MC, Lang HC (2019). Association between continuity of care and long-term mortality in Taiwanese first-ever stroke survivors: an 8-year cohort study. PLoS ONE.

[18] Uno C, Maeda K, Wakabayashi H (2020). Nutritional status change and activities of daily living in elderly pneumonia patients admitted to acute care hospital: a retrospective cohort study from the Japan Rehabilitation Nutrition Database. Nutrition.

[19] Yoshimatsu K, Nakatani H (2023). Development of a scale measuring home-visiting nurses’ attitudes toward patient safety: a cross-sectional study. BMC Nurs.

[20] Huang ZP, Huang F, Liang Q (2023). Socioeconomic factors, perceived stress, and social support effect on neonatal nurse burnout in China: a cross-sectional study. BMC Nurs.

[21] Zhang Y, Jiang J, Zhu C, Liu C, Guan C, Hu X (2022). Status and related factors of burnout among palliative nurses in China: a cross-sectional study. BMC Nurs.

[22] Wang C (2023). The real experience of patients with enterostomy receiving Internet Plus home nur-sing service:a qualitative study. J Nurs Sci.

[23] Pyo J, Lee W, Choi EY, Jang SG, Ock M (2023). Qualitative research in healthcare: necessity and characteristics. J Prev Med Public Health.

[24] Tong A, Sainsbury P, Craig J (2007). Consolidated criteria for reporting qualitative research (COREQ): a 32-item checklist for interviews and focus groups. Int J Qual Health Care.

[25] Hsieh HF, Shannon SE (2005). Three approaches to qualitative content analysis. Qual Health Res.

[26] Kondracki NL, Wellman NS, Amundson DR (2002). Content analysis: review of methods and their applications in nutrition education. J Nutr Educ Behav.

[27] Korstjens I, Moser A (2018). Series: practical guidance to qualitative research. Part 4: trustworthiness and publishing. Eur J Gen Pract.

[28] Shahrestanaki SK, Rafii F, Najafi Ghezeljeh T, Farahani MA, Majdabadi Kohne ZA (2023). Patient safety in home health care: a grounded theory study. BMC Health Serv Res.

[29] Shahriari M, Nia DH, Kalij F, Hashemi MS (2024). Challenges of home care: a qualitative study. BMC Nurs.

[30] Alsenany SA, Alharbi AA (2025). Evaluation of geriatric care competencies among nurses working in home health care in Saudi Arabia. J Gerontol Nurs.

[31] Hailemariam T, Atnafu A, Gezie LD, Tilahun B (2024). Effect of short message service reminders in improving optimal antenatal care, skilled birth attendance and postnatal care in low-and middle-income countries: a systematic review and meta-analysis. BMC Med Inform Decis Mak.

[32] Harrison JD, Greysen RS, Jacolbia R, Nguyen A, Auerbach AD (2016). Not ready, not set…discharge: patient-reported barriers to discharge readiness at an academic medical center. J Hosp Med.

[33] Kaya S, Sain Guven G, Aydan S (2018). Patients’ readiness for discharge: predictors and effects on unplanned readmissions, emergency department visits and death. J Nurs Manag.

[34] Megido I, Sela Y, Grinberg K (2023). Cost effectiveness of home care versus hospital care: a retrospective analysis. Cost Eff Resour Alloc.

[35] Goh AMY, Polacsek M, Malta S (2022). What constitutes “good” home care for people with dementia? An investigation of the views of home care service recipients and providers. BMC Geriatr.

[36] Plantinga LC, Bender AA, Urbanski M (2023). Work experiences of the interdisciplinary dialysis workforce in the United States: a cross-sectional survey. Am J Nephrol.

[37] Plantinga LC, Rickenbach F, Urbanski M (2023). Professional fulfillment, burnout, and turnover intention among US dialysis patient care technicians: a national survey. Am J Kidney Dis.

[38] Ryder M, Jacob E, Hendricks J (2020). A survey identifying leadership and research activities among nurse practitioners. Contemp Nurse.

[39] Tyler N, Hodkinson A, Planner C (2023). Transitional care interventions from hospital to community to reduce health care use and improve patient outcomes: a systematic review and network meta-analysis. JAMA Netw Open.

[40] Nantsupawat A, Kunaviktikul W, Nantsupawat R, Wichaikhum OA, Thienthong H, Poghosyan L (2017). Effects of nurse work environment on job dissatisfaction, burnout, intention to leave. Int Nurs Rev.

[41] Preziosi P, Kovner C (2023). Migrating nurses: more than addressing the U.S. nurse shortage. Policy Polit Nurs Pract.

[42] State of the world’s nursing 2020: investing in education, jobs and leadership. World Health Organization.

[43] Yu HY, Xu SH, Chen YL, Li YX, Yang QH (2022). Nurses’ perceptions regarding barriers to implementing the Internet Plus Nursing Service programme: a qualitative study. J Nurs Manag.

[44] Gao Y, Zhao H, Li X, Xie L (2022). Experiences of "Internet plus nursing service" in Chinese nurses: a meta-synthesis of qualitative studies [Article in Chinese]. J Nurs Sci.

[45] Tian Y (2024). Study on nurses’ experience of participating in "Internet+nursing service" in county hospitals [Article in Chinese]. Chin J Nurs Educ.

[46] Liu Y, Gao X, Chen H, Halimire A, Wu Y (2021). Current status and research progress of international home care modes [Article in Chinese]. Chin J Modern Nurs.

[47] Yu X, Huang Y, Liu Y (2022). Nurses’ perceptions of continuing professional development: a qualitative study. BMC Nurs.

[48] Mahat S, Rafferty AM, Vehviläinen-Julkunen K, Härkänen M (2022). Negative emotions experienced by healthcare staff following medication administration errors: a descriptive study using text-mining and content analysis of incident data. BMC Health Serv Res.

[49] Wang Y, Lu J, Ye Q (2022). Analysis of influencing factors of nurse-patient disputes based on patient characteristics: a cross-sectional study. Nurs Open.

[50] Rosen MA, DiazGranados D, Dietz AS (2018). Teamwork in healthcare: key discoveries enabling safer, high-quality care. Am Psychol.

[51] Sabone M, Mazonde P, Cainelli F (2020). Everyday ethical challenges of nurse-physician collaboration. Nurs Ethics.

